# MutationDistiller: user-driven identification of pathogenic DNA variants

**DOI:** 10.1093/nar/gkz330

**Published:** 2019-05-20

**Authors:** Daniela Hombach, Markus Schuelke, Ellen Knierim, Nadja Ehmke, Jana Marie Schwarz, Björn Fischer-Zirnsak, Dominik Seelow

**Affiliations:** 1 Berliner Institut für Gesundheitsforschung (BIH), Berlin, Germany Charité-Universitätsmedizin Berlin, corporate member of Freie Universität Berlin and Humboldt-Universität Berlin, and Berlin Institute of Health, Berlin, Germany; 2 Charité-Universitätsmedizin Berlin, Charité-BIH Centrum for Therapy and Research, Berlin, Germany; 3 NeuroCure Cluster of Excellence, Berlin, Germany; 4 Department of Neuropaediatrics, Berlin, Germany; 5 Institute of Medical Genetics and Human Genetics, Berlin, Germany; 6 Max-Planck-Institute for Molecular Genetics, RG Development & Disease, Berlin, Germany

## Abstract

MutationDistiller is a freely available online tool for user-driven analyses of Whole Exome Sequencing data. It offers a user-friendly interface aimed at clinicians and researchers, who are not necessarily bioinformaticians. MutationDistiller combines MutationTaster's pathogenicity predictions with a phenotype-based approach. Phenotypic information is not limited to symptoms included in the Human Phenotype Ontology (HPO), but may also comprise clinical diagnoses and the suspected mode of inheritance. The search can be restricted to lists of candidate genes (e.g. virtual gene panels) and by tissue-specific gene expression. The inclusion of GeneOntology (GO) and metabolic pathways facilitates the discovery of hitherto unknown disease genes. In a novel approach, we trained MutationDistiller's HPO-based prioritization on authentic genotype–phenotype sets obtained from ClinVar and found it to match or outcompete current prioritization tools in terms of accuracy. In the output, the program provides a list of potential disease mutations ordered by the likelihood of the affected genes to cause the phenotype. MutationDistiller provides links to gene-related information from various resources. It has been extensively tested by clinicians and their suggestions have been valued in many iterative cycles of revisions. The tool, a comprehensive documentation and examples are freely available at https://www.mutationdistiller.org/

## INTRODUCTION

Next Generation Sequencing has led to a large advance in the elucidation of monogenic diseases. With Whole Exome Sequencing (WES), numerous causal mutations have been found in the last decade. WES is now frequently used in research and routine clinical diagnostics. It is currently considered the most cost-effective method of genetic analysis (1).

With each WES run, tens of thousands of DNA variants have to be sifted through—a task that cannot be achieved without the aid of computer tools. The pathogenicity of a variant within protein-coding transcripts can be assessed by a number of tools, such as SIFT (2), PolyPhen2 (3) or MutationTaster (4). While the first are limited to analyzing non-synonymous single nucleotide variants (SNVs), MutationTaster also handles InDels and non-coding variants.

With an average specificity of the aforementioned tools <90% and without additional clinical information, a typical WES run produces many false positive predictions, even after filtering out common polymorphisms. In addition, most humans carry several known disease mutations in heterozygous state and even some in homozygous state (5,6).

**Figure 1.**
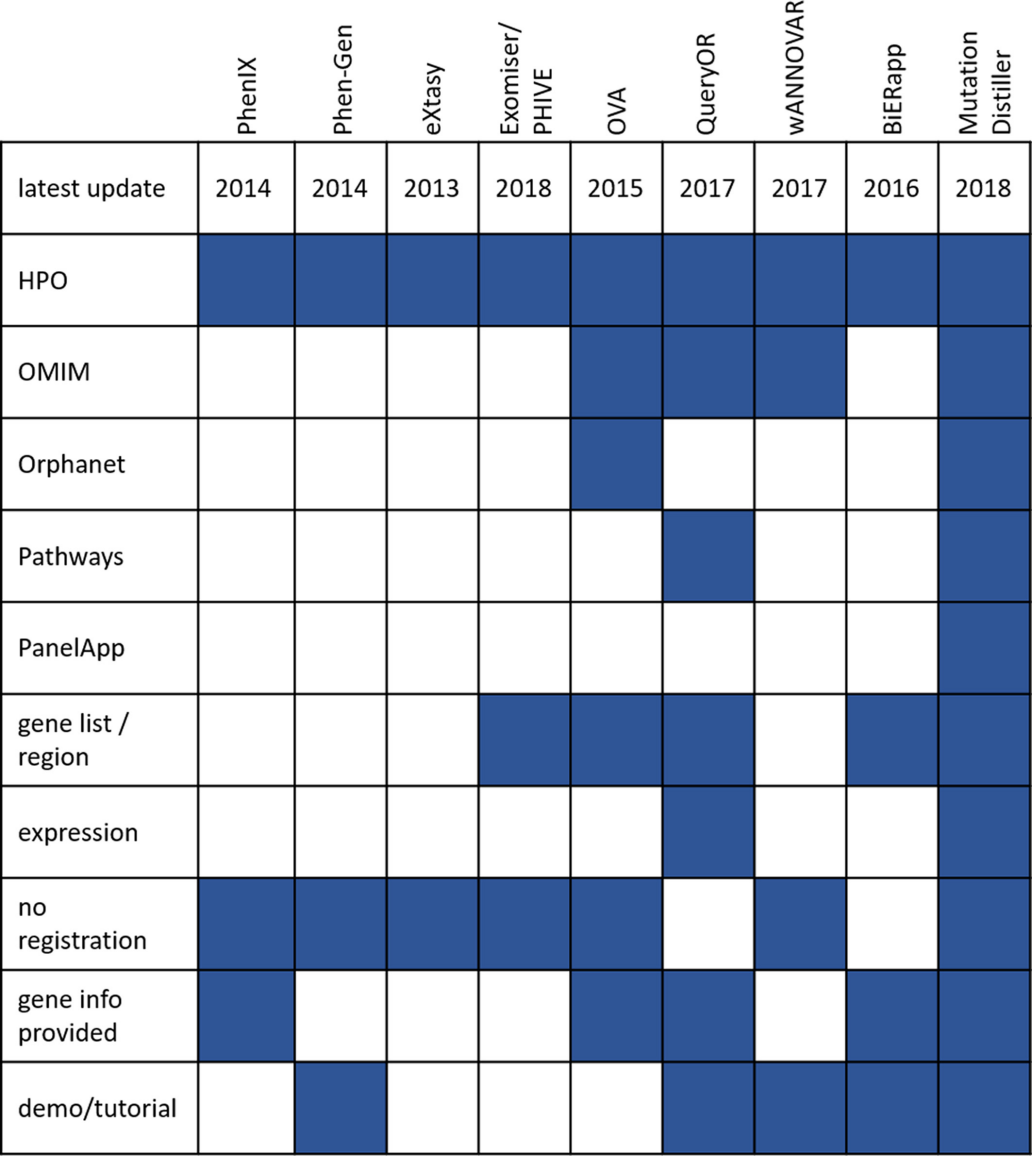
Feature comparison of current state-of-the-art prioritization tools. Blue fields indicate that the tool provides the feature.

The inclusion of further patient information allows to focus the search onto genes which are likely to be connected with the patient's symptoms. A number of tools for prioritising variants using phenotypic information exist. Phen-Gen (7), eXtasy (8), PhenIX (9) and the Exomiser (10) are based on the HPO (11) to describe the phenotype. Phevor (12) includes GO (13), the Mammalian Phenotype Ontology (14), and others, whereas ANNOVAR (15) can take free disease-related terms as input. Other tools, such as OVA (16), BiERapp (17) or QueryOR (18) are web-based frameworks which allow users to assess and re-assess in various steps and from different angles but are often not available without registration (BiERapp, QueryOR). Figure 1 gives an overview of the features of current variant prioritisation tools.

Despite the abundance of available tools, only few have found their way into routine clinical applications. Often, they are too complex for routine clinical use or do not provide enough information for users to draw meaningful conclusions from their predictions. A recent paper by Shyr *et al.* (19) stated the prime importance of usability and easy access to software for the success of sequencing projects, a need which is often not met by current tools: For instance, most tools can only accept non-synonymous SNVs and are limited to nuclear DNA, thereby excluding the mitochondrial DNA and a large number of variants from analysis. This is especially problematic for small InDels given their potential to induce frameshifts. Another major hurdle for clinical use is file size restrictions, as many web-based applications cannot handle complete WES VCF files. Moreover, missing details about a gene's function or its role in diseases or hyperlinks to external resources force the user to manually search the Internet for further information. While this might seem trivial, it is time-consuming and potentially prevents time-pressed clinicians and clinical geneticists from adopting a useful software.

Responding to the request for user-friendly prioritisation software, we have developed MutationDistiller, a tool for the analysis of WES data of patients with monogenic disorders. MutationDistiller was developed in an iterative process, taking the input of clinicians and researchers on board and aiming at creating software which adapts to the user, not vice versa. Recently, the tool has been used in over 1000 analyses per month. It is freely available at https://www.mutationdistiller.org/. The source code will be made available on request.

### Data sources

MutationDistiller integrates and combines various data sources to present the user with a list of candidate genes and variants which are most fitting for their specific case. The tool combines the pathogenicity predictions of MutationTaster (4) with the gene prioritisations of GeneDistiller (20) in a user-friendly way. MutationDistiller is capable of analyzing coding and non-coding intragenic alterations as well as mtDNA variants. The output includes key features of the predicted variant effect (such as nonsense-mediated decay) and provides hyperlinks to MutationTaster's detailed analysis of the variant.

The data sources integrated into MutationDistiller allow assessing a case from various angles, making the tool attractive to a variety of user groups: *Clinical symptoms or diagnoses* can be entered via the widely used resources HPO (11), OMIM (21) or Orphanet (22). In addition, genes known to cause diseases in mice (MGD, (23)) can be marked for highlighting and clinical symptom descriptions (HPO) of patients can be added, removed, or refined in an iterative process. *Gene function specifications* can be selected from the Gene Ontology (GO) (13), or via the pathway resources WikiPathways (24) or Reactome (25). In addition, users can filter their variants using *gene expression* data obtained from ExpressionAtlas (26), or via genetic regions, lists of candidate genes (e.g. in-house panels), or common virtual panels (11,27), Genomics England PanelApp, (https://panelapp.genomicsengland.co.uk/). MutationDistiller also contains the ACMG SF v2.0 actionable genes panel, a list of genes of medical relevance published by the American College of Medical Genetics and Genomics (ACMG) (28). Disease-causing mutations in these genes are considered to be ‘actionable’ and may be revealed to patients in case they had opted to be informed about incidental findings.

Moreover, we integrated ClinVar (29) to allow the identification of known disease-causing mutations and data from the Thousand Genomes Project (1000G) (30) as well as ExAC (31) to filter for polymorphisms.

### The MutationDistiller workflow

In a first step, users upload a VCF file and can give and refine phenotypic patient information such as symptoms or diagnoses to obtain a prioritised list of candidate variants. The uploaded variants can be filtered for genomic region, candidate genes, homozygosity, coverage and presence in polymorphisms databases. The remaining alterations are then analysed by MutationTaster for their effect on their gene products and the results for each of them are permanently stored in our database.

The upload of a typical WES VCF file with 50 000 variants takes ∼5 min or considerably less if the file contains a high number of variants already known to our database. In our database, we store the results for each variant permanently but not the genotypes of a single sample (to comply with privacy requirements). This approach saves run-time for subsequent re-submission of the same variant in a later project. Genotypes and coverage from a VCF file are stored separately in a sample-specific table which can be deleted by the user at any time.

In addition, upload filters (e.g. restriction to a certain genomic region or a list of candidate genes) can save upload times. Upon completion of the upload, users get notified via email (if provided) and are redirected to the analysis page. Subsequent (re-)analyses require mere milliseconds of loading time. Access to the project is only granted via a dedicated access key in the URL. To account for data protection, all projects can be removed manually. If not deleted by the user, data will be retained for at least 4 weeks.

As mentioned, users can process a wealth of patient information with MutationDistiller. In addition to the clinical focus for detection of variants in known disease genes (via OMIM, HPO, and Orphanet), inclusion of other domains such as gene function (GO, molecular pathways) or gene expression allow discovery of variants in hitherto unknown genes. The focus on molecular pathways may be of service in cases where laboratory results point towards disrupted molecular pathways (e.g. a defect in β-oxidation, or in TGF-β signalling), while expression data might be of interest if a specific tissue is known to be affected (e.g. liver dysfunction). Moreover, users can filter their data according to variant type (e.g. splice-site variant vs. premature stop-codon) and by the suspected mode of inheritance.

As the plethora of data entry options might be overwhelming for first-time users, we provide a number of dedicated user modes for different interest groups, limiting the amount of choices users are exposed to upon first access to our web site.

From the submitted variants and data, MutationDistiller presents a prioritized list of the most likely candidate variants and a wealth of information on the variants and their genes. In addition, we provide hyperlinks to relevant resources, saving users from having to search the Internet. To prevent users from being flooded by too much information, MutationDistiller displays a downloadable summary table containing the most crucial data on the candidate variants and their genes at the top of the output page, followed by more detailed information for each gene below. Hyperlinks to the result or personalised query pages can be generated and shared to facilitate collaborative projects. To allow iterative analyses, parameters can be updated and altered on-the-fly at various stages. For instance, HPO terms can be retrospectively added to or removed from analysis on the result page to fine-tune the search.

### MutationDistiller score

MutationDistiller bases its prioritization on a score representing how well a variant and its gene match the user criteria. The submitted variants are not scored by severity as MutationTaster's predictions are of Boolean nature. Instead, they are grouped into different classes according to the severity of the predicted effect (e.g. frameshift versus non-coding), allowing users to focus on certain types of alterations (e.g. stop-codon inducing).

For scoring, MutationDistiller balances the various data domains against each other: Each of them enters into the score with a weight representing its assumed biological relevance. In addition to the user-defined domains, we also score known disease-causing mutations (listed as *pathogenic* in ClinVar). Due to the lack of available phenotypic data for test mutations, such weights could not be optimised by statistical analysis. For the HPO symptoms, however, we were able to develop a dynamic weighted score.

In this scoring system, the base score of a HPO term is determined by its information content (IC), one of the standard methods for ontologies (32). This IC is represented by the negative logarithm of the number of genes annotated with a given term divided by the total number of genes annotated with any HPO term (currently 2233). In addition, we allocate different weights to direct HPO matches versus ancestors or descendants to limit the impact of phenotyping errors.

In a novel approach, we optimized these weights on a set consisting of known disease mutations from ClinVar linked with HPO terms: We obtained all *pathogenic* ClinVar entries with at least two HPO terms; a total of 188 cases linked with 142 different genes. Please refer to our web site for this test set. We spiked these mutations into the HG00377 exome from the 1000 Genomes Project and sent them, together with the associated HPO terms, to MutationDistiller. Subsequently, we iterated through a range of weight combinations (245 combinations in total) for direct, ancestor and descendant matches and compared the results. If the disease mutation was found, we then observed the distribution of the ranks given to the genes containing the disease mutation across all weight combinations. We only regarded the first 100 ranks, denoting any cases beyond that as ‘not found’. Genes with the exact same score were given the same rank.

For each of the various weight-combinations, a relatively high number of cases (at least 20%) could not be solved, indicating that the phenotypes entered into ClinVar are not always identical with the phenotypes annotated with the disease genes in the HPO, which corresponds to a real-life situation in clinical diagnostics. We assessed the resulting weight combinations to find a balanced solution that would consistently rank the causative gene highly while showing a low rate of unsolved cases; and after careful consideration we settled for a weight of 5 for direct matches, 0.05 for descendants and 2 for ancestor terms. We chose this approach rather than dynamically searching for the optimal weight distribution to avoid overfitting on this relatively small data set. A summary of the tested weight combinations and their results can be found on our web site.

### Comparison with state-of-the-art tools

To validate MutationDistiller's HPO-based prioritisations, we compared it to other tools sharing similar properties: In our test, we included widely used and freely available functional state-of-the-art tools which do not require any local software installation or user login, can work with single patient VCF files and offer HPO-based prioritisations. We found three different algorithms fulfilling these criteria: the PhenIX (9) and HiPhive (33) algorithms incorporated into Exomiser (34) (version exomiser-cli-10.0.1), and eXtasy (8). (version 2013-07-04) For our analyses, we used default settings for all algorithms, which is what an untrained user would be expected to do. Despite our aim to only include web-based software, we had to rely on locally installed versions as the online tools were not working reliably or fast enough for our purposes.

We tested the software on a set of 101 solved patients from the Charité who had given informed consent for research use. These instances of rare, early-onset Mendelian disorders were provided by clinicians and researchers working in the Department of Neuropediatrics and the Institute of Medical Genetics and Human Genetics. We used newly found disease mutations which were not yet included in ClinVar, together with the HPO symptoms assigned to the patient and information on the expected mode of inheritance (if available). The set included a range of disorders and various types of mutations as well as compound heterozygous cases. We spiked the known causative variant for each case into the same 1000G exome VCF file used for optimization of MutationDistiller (HG00377).

We then sent the resulting VCF files, the HPO identifiers, and mode of inheritance information submitted by the clinicians to the different tools. For MutationDistiller, we used the HPO weight settings determined in the optimization procedure described above. The tools included into this comparison do not provide a score for known pathogenic variants, which is why we decided not to take into account the MutationDistiller's ClinVar score at this stage.

As in the optimization step, we recorded the ranks allocated to the genes containing the index mutation, capping at rank 100. For the four tools or algorithms, we then compared the distribution of ranks for the index genes.

To assess the prioritization of Exomiser, we used its ‘Exomiser gene pheno score’, which does not include the variant prediction but only the phenotypic assessment of the gene. As the eXtasy algorithm is not capable of working with all HPO terms, we removed for this tool the terms not found in eXtasy's database from our set. This limited our set for eXtasy analysis to 88 cases. Moreover, eXtasy's entry options are limited to 10 HPO symptoms per case. In the 7 cases with more than 10 HPO terms, we thus randomly removed symptoms to reach only 10 terms. Moreover, for eXtasy, we had to distinguish between cases in which only one HPO term was used for analysis and cases with more than one term. In cases with a single HPO term, we ranked the files by the result score; in combined cases by the provided statistical score as the program outputs a result score for each HPO term separately.

**Figure 2.**
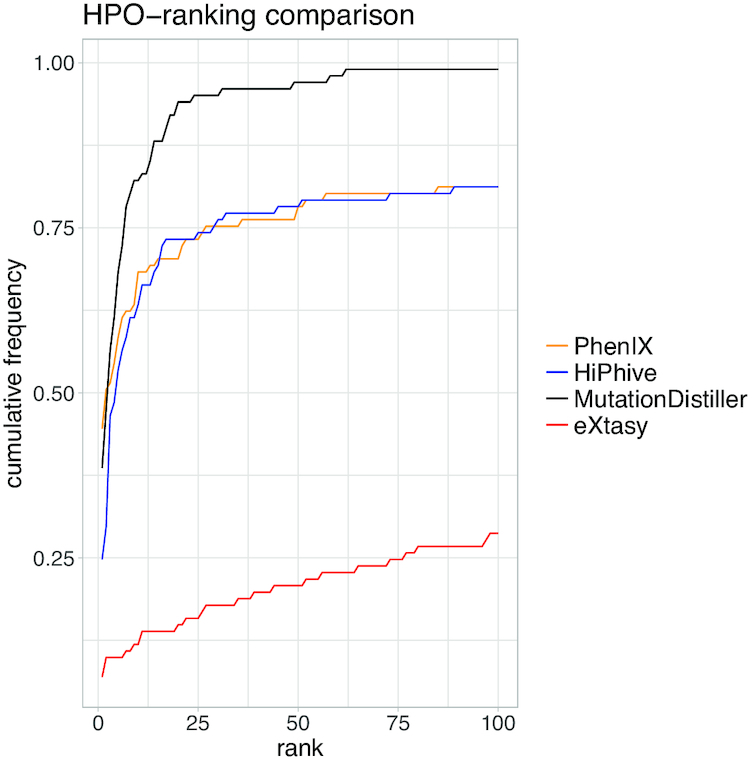
Cumulative rank distributions for the HPO-based detection of known disease mutations in a set of 101 patient files for MutationDistiller (black), PhenIX (orange), HiPhive (blue) and eXtasy (red). For each tool, we depict the accuracy as the cumulative percentage of indicated disease genes ranked within each rank group (top 1 to top 100).

When comparing the cumulative ranks allocated to the genes of interest, we found that eXtasy failed in many of the provided cases: For reasons mentioned above, the analysis was limited to 88 of the 101 test cases. Of these, eXtasy listed less than 30% of the causative alterations within the first 100 ranks, which might be due to the fact that the program was last updated in 2013 and hence cannot profit from large parts of the regularly updated HPO. HiPhive and PhenIX detected the causative gene within the top 100 positions in the majority of cases. However, MutationDistiller was capable of solving considerably more cases than the other tools (99.0% for MutationDistiller, 81.2 % for PhenIX and HiPhive, 28.7% for eXtasy). The accuracy for the first rank was slightly higher in PhenIX (44.5%) than in MutationDistiller (38.6%) and HiPhive (24.7%) and considerably higher than in eXtasy (6.9%). For the first 10 or 20 ranks, MutationDistiller's accuracy clearly outperformed the other tools (10 – MutationDistiller: 82.2%, PhenIX: 68.3%, HiPhive: 63.3%, eXtasy: 11.9%; 20 – MutationDistiller: 94.1%, HiPhive: 73.3%, PhenIX: 70.3%, eXtasy: 14.9%). Figure 2 displays the cumulative rank distributions for the tested algorithms. A more in-depth description of the comparison together with the results can be found on our web site and in the supplement.

## DISCUSSION AND OUTLOOK

MutationDistiller is a one-stop-shop for physicians, human geneticists, and genetic counselors. It offers phenotype-based WES analyses using diverse patient data. With our tool, we put emphasis on adaptability and user-friendliness, thus aiming to answer to the urgent request for user-focused WES data analysis (19). During our development process, we have focused on an iterative approach, taking clinicians’ and researchers’ needs into account at any stage.

A major problem in describing clinical phenotypes are errors and gaps, which might depend on the experience of the phenotyping clinician. During our optimisation and testing procedures, we have noticed that in several cases, two clinicians would assign different HPO terms to the same patient. This may be due to their personal and independent evaluation, or simply because they examined the patient at different time points or under different circumstances. To allow for these naturally occurring inconsistencies, we decided to train and optimize our HPO score neither on artificial data sets, nor on HPO symptoms assigned to the gene in databases or self-selected HPO symptoms, but on curated ClinVar phenotype collections. These were originally derived from real patients in a clinical setting and enabled us to base our prioritisations on authentic data.

Using this set, we developed a dynamic HPO scoring system which can account for gaps and errors in the phenotype assignments by incorporating ancestor and descendant terms. We demonstrated that this HPO score is capable of competing with state-of-the-art prioritization tools when detecting causative mutations in routine clinical cases (see Figure 2). In our comparison, we have restricted ourselves to only use HPO and mode of inheritance information to stay on the same level with the capabilities of the other tools. This means that in real life, MutationDistiller results can be expected to be even better for known disease mutations. We believe that both the novel approach of using of authentic training cases and the balancing of phenotype gaps play a role in MutationDistiller's prioritization success. By mirroring a clinical setting, we were able to create a tool which is well adjusted for use in real-life clinical cases. While our training and testing sets are currently comparatively small due to the lack of openly available genotype–phenotype connections, we are planning to train and test our tool on larger sets as they arise in the future.

In addition to HPO-centered analyses, we decided to offer a wide range of data entry and filtering options as this opens the tool for users from various backgrounds. Besides, this allows users to detect mutations located in hitherto unknown disease genes, which is an advantage in comparison to other tools. While we are presently not able to optimize these features in a statistically sound way, we are planning to change this as more data informative sets arise.

Similarly, we are planning to incorporate a number of features if new fitting data resources are developed: As MutationDistiller bases its predictions on MutationTaster, it can currently only detect variants located in protein-coding transcripts. This includes both coding and non-coding alterations and is thus already more comprehensive than a wide range of many other tools. The limitation is due to the fact that so far only few disease mutations outside of protein-coding genes are known. We expect this to change in the future, with the advent of Whole Genome Sequencing and CRISPR/Cas9 for validation of the effect of extragenic variants. We are planning to update MutationDistiller accordingly to prepare for the age of Whole Genome Sequencing.

In addition, we are currently working on a number of further developments suggested by our users. For instance, we are in the process of implementing compatibility for trio or family VCF analyses, allowing users to compare an affected patient's variants to unaffected family members or controls. Another feature proposed by our users is the adaptation of MutationTaster's predictions to the ACMG guidelines for sequence variant interpretation (35). While MutationTaster's predictions largely follow these suggestions, we have not yet incorporated them explicitly. However, we intend to update this in the near future, which would allow MutationDistiller to also fulfill these criteria and facilitate the authoring of clinical reports. Moreover and as suggested by our users, the integration of data from gnomAD (31) to recognize and filter polymorphisms is already under development.

MutationDistiller is currently based on GRCh37 (hg19) which is still the common genome build for WES, Thus far, we have not received any requests for GRCh38 and decided that offering parallel genome builds is not necessary at this stage. However, we are planning to implement this in the future, should the need arise.

## CONCLUSION

With MutationDistiller, we present a tool which provides fast, convenient and reliable analyses of WES data is highly adaptable to different users’ requirements. We optimized and validated MutationDistiller's HPO scoring system on real-life clinical data and demonstrated that it can match or outcompete similar approaches. With a total of >11 000 analyses and >1000 analyses per month, MutationDistiller has already found its way into the clinic. With growing numbers of routine WES applications, we are convinced that its user group will see further increases in the future.

## SOFTWARE IMPLEMENTATION AND DATA INTEGRATION

MutationDistiller runs on a 48-core system with 512 GB RAM under Linux (CentOS 6). All data are physically integrated and stored in a PostgreSQL 9.5 database. Jobs are scheduled by TORQUE (version 4.2). Program scripts are written in Perl (5.10) and run in an Apache 2.2 web server. All user interfaces are written in HTML with JavaScript functions and are developed for the Firefox browser. Additional testing was conducted on Google Chrome and Safari.

The source code will be made available on request.

## Supplementary Material

gkz330_Supplemental_Files
