# Supplementary material for: MutationDistiller: user-driven identification of pathogenic DNA variants
Source: Nucleic Acids Res. 2019 May 20;47(W1):W114–20. doi: 10.1093/nar/gkz330 (PMC6602447; doi:10.1093/nar/gkz330)
Supplement: gkz330_Supplemental_Files [file gkz330_supplemental_files.zip › Supplement_MD_FINAL.pdf]

MutationDistiller – user-driven identification of pathogenic DNA variants

Daniela Hombach*[1,2,3], Markus Schuelke[3,4], Ellen Knierim[3,4], Nadja Ehmke[5], Jana Marie Schwarz[2,3,4], Björn Fischer-Zirnsak[5,6] & Dominik Seelow[1,2]

[1] Berliner Institut für Gesundheitsforschung (BIH), Berlin, Germany

Charité-Universitätsmedizin Berlin, corporate member of Freie Universität Berlin and Humboldt-Universität Berlin, and Berlin Institute of Health [2] Charité-Universitätsmedizin Berlin, Charité-BIH Centrum for Therapy and Research, [3] NeuroCure Cluster of Excellence, [4] Department of Neuropaediatrics, [5] Institute of Medical Genetics and Human Genetics

[6] Max-Planck-Institute for Molecular Genetics, RG Development & Disease, Berlin, Germany

* corresponding author: daniela.hombach@charite.de

URL: https://www.mutationdistiller.org/

Supplementary Material

Optimisation iterations

We optimised MutationDistiller's HPO weights on a set consisting of known disease mutations from ClinVar (https://www.ncbi.nlm.nih.gov/clinvar/) linked with HPO terms: We obtained all pathogenic ClinVar entries with at least two HPO terms; a total of 188 cases linked with 142 different genes. Please refer to our web site for this test set. We spiked these mutations into the HG00377 exome from the 1000 Genomes Project (http://www.internationalgenome.org/) and sent them, together with the associated HPO terms, to MutationDistiller. Subsequently, we iterated through a range of weight combinations (245 combinations in total) for direct, ancestor and descendant matches and compared the results. If the disease mutation was found, we then observed the distribution of the ranks given to the genes containing the disease mutation across all weight combinations. We only regarded the first 100 ranks, denoting any cases beyond that as *not found*. Genes with the exact same score were given the same rank.

A VCF file containing all the ClinVar variants we used, together with their HPO symptoms, is also available as Supplementary Material.

The results for all iterations through 245 different weight combinations for direct HPO matches, ancestor and descendant term matches are listed in the table below.

Column titles:

- **index**: number of the combination of weights (model number)
- **direct/ancestor/descendant weight**: weight assigned to the different match types
- **first rank**: number of correct disease genes allocated to the first rank
- **rank 1-5**: number of correct disease genes allocated to ranks 1-5
- **rank >10**: number of correct disease genes allocated to ranks greater than 10
- **not found**: number of unsolved cases, i.e. correct disease gene ranked >100

We chose this combination as it is balanced and consistently ranks the causative gene highly while showing a low rate of unsolved cases. The table is sorted by the amount of cases which were not solved (column 'not found') as first parameter and by the mean rank as second parameter.

All tables are also available as TSV files on our website.

index	direct weight	ancestor weight	descendant weight	first rank	rank 1-5	rank >10	mean rank	not found
145	1.00	2.00	0.50	7	74	37	9.5	42
31	0.20	0.50	0.10	6	65	38	10.1	42
38	0.20	1.00	0.10	3	49	48	11.5	42
45	0.20	2.00	0.10	3	46	52	11.9	42
* 210	5.00	0.05	2.00	70	108	26	5.8	43
224	5.00	0.20	2.00	70	109	26	5.9	43
217	5.00	0.10	2.00	70	108	26	5.9	43
160	2.00	0.05	1.00	67	108	26	6	43
60	0.50	0.05	0.20	70	107	24	6	43
231	5.00	0.50	2.00	70	107	24	6	43
110	1.00	0.05	0.50	67	108	26	6	43
167	2.00	0.10	1.00	67	108	26	6	43
117	1.00	0.10	0.50	67	107	26	6.1	43
174	2.00	0.20	1.00	67	107	26	6.1	43
67	0.50	0.10	0.20	69	106	24	6.2	43
238	5.00	1.00	2.00	69	106	24	6.2	43
124	1.00	0.20	0.50	66	106	25	6.2	43
111	1.00	0.05	1.00	56	104	27	6.3	43
168	2.00	0.10	2.00	56	104	27	6.3	43
10	0.20	0.05	0.10	66	105	25	6.3	43
181	2.00	0.50	1.00	66	105	25	6.3	43
161	2.00	0.05	2.00	56	103	28	6.3	43
61	0.50	0.05	0.50	56	105	27	6.4	43
118	1.00	0.10	1.00	56	105	27	6.4	43
175	2.00	0.20	2.00	56	105	27	6.4	43
11	0.20	0.05	0.20	56	104	27	6.5	43
68	0.50	0.10	0.50	56	103	28	6.5	43
125	1.00	0.20	1.00	56	103	28	6.5	43

*combination used in MutationDistiller (version 2019-04-09)

182	2.00	0.50	2.00	56	104	27	6.5	43
17	0.20	0.10	0.10	61	99	25	6.6	43
131	1.00	0.50	0.50	61	99	25	6.6	43
188	2.00	1.00	1.00	61	99	25	6.6	43
75	0.50	0.20	0.50	55	101	26	6.7	43
112	1.00	0.05	2.00	42	102	27	6.8	43
18	0.20	0.10	0.20	55	98	27	6.8	43
132	1.00	0.50	1.00	55	98	27	6.8	43
189	2.00	1.00	2.00	55	98	27	6.8	43
62	0.50	0.05	1.00	42	100	28	6.9	43
119	1.00	0.10	2.00	42	100	28	6.9	43
24	0.20	0.20	0.10	39	90	27	7.4	43
138	1.00	1.00	0.50	39	90	27	7.4	43
195	2.00	2.00	1.00	39	90	27	7.4	43
25	0.20	0.20	0.20	37	90	28	7.6	43
82	0.50	0.50	0.50	37	90	28	7.6	43
139	1.00	1.00	1.00	37	90	28	7.6	43
196	2.00	2.00	2.00	37	90	28	7.6	43
63	0.50	0.05	2.00	37	88	28	7.6	43
89	0.50	1.00	0.50	8	69	32	9	43
146	1.00	2.00	1.00	8	69	32	9	43
88	0.50	1.00	0.20	8	75	36	9.1	43
90	0.50	1.00	1.00	11	67	35	9.2	43
147	1.00	2.00	2.00	11	67	35	9.2	43
87	0.50	1.00	0.10	8	75	38	9.2	43
144	1.00	2.00	0.20	8	75	38	9.2	43
142	1.00	2.00	0.05	8	74	37	9.3	43
86	0.50	1.00	0.05	8	74	38	9.3	43
143	1.00	2.00	0.10	8	74	38	9.3	43
32	0.20	0.50	0.20	6	60	34	9.6	43
91	0.50	1.00	2.00	14	62	39	9.6	43
33	0.20	0.50	0.50	9	57	38	9.7	43
30	0.20	0.50	0.05	6	66	39	9.8	43
34	0.20	0.50	1.00	13	53	43	10.1	43
35	0.20	0.50	2.00	15	54	42	10.2	43
93	0.50	2.00	0.05	3	51	45	10.7	43
94	0.50	2.00	0.10	3	51	46	10.7	43
97	0.50	2.00	1.00	3	46	47	10.7	43
95	0.50	2.00	0.20	3	51	44	10.8	43
96	0.50	2.00	0.50	3	47	44	10.8	43
98	0.50	2.00	2.00	8	45	52	10.8	43
37	0.20	1.00	0.05	3	50	47	11	43
39	0.20	1.00	0.20	3	45	47	11	43
40	0.20	1.00	0.50	3	44	54	11.2	43
41	0.20	1.00	1.00	7	42	55	11.2	43
42	0.20	1.00	2.00	12	42	55	11.2	43

44	0.20	2.00	0.05	3	47	50	11.5	43
46	0.20	2.00	0.20	3	42	52	11.5	43
49	0.20	2.00	2.00	6	38	60	11.7	43
47	0.20	2.00	0.50	3	40	57	11.7	43
48	0.20	2.00	1.00	3	41	61	12.1	43
159	2.00	0.05	0.50	69	110	23	5.5	44
209	5.00	0.05	1.00	69	108	23	5.5	44
216	5.00	0.10	1.00	69	108	23	5.5	44
208	5.00	0.05	0.50	65	106	24	5.5	44
223	5.00	0.20	1.00	69	107	23	5.5	44
109	1.00	0.05	0.20	69	107	23	5.5	44
166	2.00	0.10	0.50	69	108	23	5.5	44
207	5.00	0.05	0.20	63	106	24	5.5	44
158	2.00	0.05	0.20	65	106	24	5.5	44
215	5.00	0.10	0.50	65	106	24	5.5	44
222	5.00	0.20	0.50	64	106	25	5.6	44
173	2.00	0.20	0.50	68	108	24	5.6	44
214	5.00	0.10	0.20	62	106	25	5.6	44
157	2.00	0.05	0.10	63	106	25	5.6	44
108	1.00	0.05	0.10	64	106	25	5.6	44
165	2.00	0.10	0.20	64	106	25	5.6	44
107	1.00	0.05	0.05	62	104	24	5.6	44
164	2.00	0.10	0.10	62	104	24	5.6	44
206	5.00	0.05	0.10	62	106	25	5.6	44
221	5.00	0.20	0.20	61	104	24	5.6	44
156	2.00	0.05	0.05	61	104	24	5.7	44
220	5.00	0.20	0.10	61	102	24	5.7	44
59	0.50	0.05	0.10	66	107	25	5.7	44
116	1.00	0.10	0.20	66	107	25	5.7	44
230	5.00	0.50	1.00	66	107	25	5.7	44
213	5.00	0.10	0.10	61	104	24	5.7	44
163	2.00	0.10	0.05	61	102	24	5.7	44
205	5.00	0.05	0.05	61	104	24	5.7	44
58	0.50	0.05	0.05	63	103	25	5.7	44
115	1.00	0.10	0.10	63	103	25	5.7	44
172	2.00	0.20	0.20	63	103	25	5.7	44
229	5.00	0.50	0.50	63	103	25	5.7	44
212	5.00	0.10	0.05	61	103	24	5.7	44
219	5.00	0.20	0.05	61	102	23	5.7	44
114	1.00	0.10	0.05	62	102	25	5.7	44
171	2.00	0.20	0.10	62	102	25	5.7	44
228	5.00	0.50	0.20	62	102	24	5.7	44
227	5.00	0.50	0.10	62	102	24	5.8	44
170	2.00	0.20	0.05	62	102	24	5.8	44
65	0.50	0.10	0.05	64	102	24	5.8	44
122	1.00	0.20	0.10	64	102	24	5.8	44

236	5.00	1.00	0.50	64	102	24	5.8	44
235	5.00	1.00	0.20	64	103	24	5.8	44
66	0.50	0.10	0.10	65	103	23	5.8	44
123	1.00	0.20	0.20	65	103	23	5.8	44
178	2.00	0.50	0.10	65	103	22	5.8	44
237	5.00	1.00	1.00	65	103	23	5.8	44
226	5.00	0.50	0.05	61	102	24	5.8	44
121	1.00	0.20	0.05	64	102	24	5.8	44
179	2.00	0.50	0.20	65	103	22	5.8	44
9	0.20	0.05	0.05	66	104	23	5.8	44
180	2.00	0.50	0.50	66	104	23	5.8	44
177	2.00	0.50	0.05	65	103	22	5.8	44
234	5.00	1.00	0.10	64	103	24	5.9	44
233	5.00	1.00	0.05	64	103	25	5.9	44
72	0.50	0.20	0.05	63	99	22	6	44
243	5.00	2.00	0.50	63	99	22	6	44
74	0.50	0.20	0.20	64	100	23	6	44
245	5.00	2.00	2.00	64	100	23	6	44
73	0.50	0.20	0.10	63	99	23	6	44
244	5.00	2.00	1.00	63	99	23	6	44
242	5.00	2.00	0.20	63	99	24	6.1	44
16	0.20	0.10	0.05	61	99	23	6.1	44
187	2.00	1.00	0.50	61	99	23	6.1	44
241	5.00	2.00	0.10	63	99	24	6.1	44
240	5.00	2.00	0.05	63	99	24	6.2	44
130	1.00	0.50	0.20	61	99	23	6.2	44
129	1.00	0.50	0.10	61	100	25	6.2	44
186	2.00	1.00	0.20	61	100	25	6.2	44
128	1.00	0.50	0.05	61	100	25	6.2	44
185	2.00	1.00	0.10	61	100	25	6.2	44
184	2.00	1.00	0.05	61	100	26	6.3	44
69	0.50	0.10	1.00	42	99	27	6.4	44
126	1.00	0.20	2.00	42	99	27	6.4	44
76	0.50	0.20	1.00	41	98	26	6.5	44
133	1.00	0.50	2.00	41	96	25	6.6	44
12	0.20	0.05	0.50	41	96	27	6.6	44
19	0.20	0.10	0.50	41	93	26	6.9	44
81	0.50	0.50	0.20	39	93	27	7	44
23	0.20	0.20	0.05	39	94	28	7.1	44
194	2.00	2.00	0.50	39	94	28	7.1	44
80	0.50	0.50	0.10	39	92	28	7.1	44
137	1.00	1.00	0.20	39	92	28	7.1	44
79	0.50	0.50	0.05	38	92	30	7.1	44
136	1.00	1.00	0.10	38	92	30	7.1	44
193	2.00	2.00	0.20	38	92	30	7.1	44
135	1.00	1.00	0.05	36	92	30	7.2	44

192	2.00	2.00	0.10	36	92	30	7.2	44
70	0.50	0.10	2.00	37	87	27	7.2	44
77	0.50	0.20	2.00	38	87	26	7.2	44
191	2.00	2.00	0.05	35	92	30	7.2	44
83	0.50	0.50	1.00	31	82	28	7.3	44
140	1.00	1.00	2.00	31	82	28	7.3	44
13	0.20	0.05	1.00	35	85	29	7.4	44
20	0.20	0.10	1.00	36	83	26	7.5	44
26	0.20	0.20	0.50	30	76	28	7.5	44
14	0.20	0.05	2.00	34	80	31	7.6	44
21	0.20	0.10	2.00	34	78	29	7.8	44
84	0.50	0.50	2.00	29	72	30	7.9	44
27	0.20	0.20	1.00	28	70	30	8.1	44
28	0.20	0.20	2.00	26	69	32	8.3	44
85	0.50	1.00	0.00	8	74	29	8.2	48
141	1.00	2.00	0.00	8	74	29	8.2	48
29	0.20	0.50	0.00	6	66	31	8.6	48
92	0.50	2.00	0.00	3	52	36	9.5	48
36	0.20	1.00	0.00	3	51	37	9.7	48
43	0.20	2.00	0.00	3	48	40	10	48
211	5.00	0.10	0.00	61	104	14	4.5	49
204	5.00	0.05	0.00	61	104	15	4.5	49
218	5.00	0.20	0.00	61	103	14	4.5	49
106	1.00	0.05	0.00	61	103	14	4.5	49
155	2.00	0.05	0.00	61	103	14	4.5	49
162	2.00	0.10	0.00	61	103	14	4.5	49
57	0.50	0.05	0.00	61	103	15	4.6	49
113	1.00	0.10	0.00	61	103	15	4.6	49
169	2.00	0.20	0.00	61	103	15	4.6	49
225	5.00	0.50	0.00	61	103	15	4.6	49
64	0.50	0.10	0.00	64	104	17	4.7	49
120	1.00	0.20	0.00	64	104	17	4.7	49
232	5.00	1.00	0.00	64	104	17	4.7	49
8	0.20	0.05	0.00	65	104	16	4.7	49
176	2.00	0.50	0.00	65	104	16	4.7	49
71	0.50	0.20	0.00	63	99	18	4.9	49
239	5.00	2.00	0.00	63	99	18	4.9	49
15	0.20	0.10	0.00	61	100	20	5.1	49
127	1.00	0.50	0.00	61	100	20	5.1	49
183	2.00	1.00	0.00	61	100	20	5.1	49
22	0.20	0.20	0.00	35	92	23	6	49
78	0.50	0.50	0.00	35	92	23	6	49
134	1.00	1.00	0.00	35	92	23	6	49
190	2.00	2.00	0.00	35	92	23	6	49
53	0.50	0.00	0.20	74	105	19	4.8	53
203	5.00	0.00	2.00	74	105	19	4.8	53

3	0.20	0.00	0.10	71	106	20	4.9	53
103	1.00	0.00	0.50	71	106	20	4.9	53
153	2.00	0.00	1.00	71	106	20	4.9	53
4	0.20	0.00	0.20	58	103	23	5.4	53
54	0.50	0.00	0.50	58	103	23	5.4	53
104	1.00	0.00	1.00	58	103	23	5.4	53
154	2.00	0.00	2.00	58	103	23	5.4	53
55	0.50	0.00	1.00	43	100	21	5.9	53
105	1.00	0.00	2.00	43	100	21	5.9	53
5	0.20	0.00	0.50	42	96	23	6.2	53
56	0.50	0.00	2.00	38	92	23	6.6	53
6	0.20	0.00	1.00	36	90	24	6.8	53
7	0.20	0.00	2.00	35	86	25	7	53
52	0.50	0.00	0.10	73	105	16	4.3	54
102	1.00	0.00	0.20	73	105	16	4.3	54
202	5.00	0.00	1.00	73	105	16	4.3	54
100	1.00	0.00	0.05	68	104	16	4.3	54
150	2.00	0.00	0.10	68	104	16	4.3	54
51	0.50	0.00	0.05	70	104	16	4.3	54
101	1.00	0.00	0.10	70	104	16	4.3	54
151	2.00	0.00	0.20	70	104	16	4.3	54
201	5.00	0.00	0.50	70	104	16	4.3	54
200	5.00	0.00	0.20	67	104	16	4.3	54
2	0.20	0.00	0.05	73	106	16	4.3	54
152	2.00	0.00	0.50	73	106	16	4.3	54
199	5.00	0.00	0.10	67	104	16	4.3	54
149	2.00	0.00	0.05	67	104	16	4.3	54
198	5.00	0.00	0.05	67	103	16	4.4	54
1	0.20	0.00	0.00	65	104	6	3	63
50	0.50	0.00	0.00	65	104	6	3	63
99	1.00	0.00	0.00	65	104	6	3	63
148	2.00	0.00	0.00	65	104	6	3	63
197	5.00	0.00	0.00	65	104	6	3	63

Comparison with other tools

To validate MutationDistiller's HPO-based prioritisations, we compared it to other tools sharing similar properties: In our test, we included widely used and freely available functional state-of-the-art tools which do not require any software installation or user login, can analyse single patient VCF files, and offer HPO-based prioritisations. We found three different algorithms fulfilling these criteria: eXtasy (http://extasy.esat.kuleuven.be/, obtained from https://github.com/asifrim/eXtasy, version 2014-02-19) and the PhenIX (http://compbio.charite.de/PhenIX/) and HiPhive algorithms incorporated into Exomiser (https://www.sanger.ac.uk/science/tools/exomiser, version exomiser-cli-10.0.1). For our analyses, we used default settings for all algorithms, which is what an untrained user would be expected to do. For each of the algorithms, we had to rely on locally installed versions as the online tools were not working reliably or fast enough for our purposes. We tested the software on a set of 101 solved patient cases from the Charité Berlin. These instances of rare, early-onset Mendelian disorders were provided by clinicians and researchers working in the Department of Neuropaediatrics and the Institute of Medical Genetics and Human Genetics. We used newly found disease mutations which were not yet included in ClinVar, together with the HPO symptoms assigned to the patient and information on the expected mode of inheritance (if available). The set included a range of disorders and various types of mutations as well as compound heterozygous cases. We spiked the known causative variant for each case into the same 1000G exome VCF file used for optimisation of MutationDistiller (HG00377). As the eXtasy algorithm is not capable of working with all HPO terms, we removed for this tool the terms not found in eXtasy's database from our set. This limited our set for eXtasy analysis to 88 cases. Moreover, eXtasy's entry options are limited to 10 HPO symptoms per case. In the 7 cases with more than 10 HPO terms, we thus randomly removed symptoms to reach only 10 terms. We then sent the resulting VCF files, the HPO identifiers and mode of inheritance information submitted by the clinicians to the different tools. For MutationDistiller, we used the HPO weight settings determined in the optimisation procedure described above. The tools included into this comparison do not provide a score for known pathogenic variants, which is why we decided not to take into account MutationDistiller's ClinVar score at this stage.

As in the optimisation step, we recorded the ranks allocated to the genes containing the index mutation, capping at rank 100. For the four tools or algorithms, we then compared the distribution of ranks for the index genes.

Exomiser: To assess the prioritisation of Exomiser, we used its *Exomiser gene pheno score*, which does not include the variant prediction but only the phenotypic assessment of the gene.

eXtasy: For eXtasy, we had to distinguish between cases in which only one HPO term was used for analysis and cases with more than one term. In cases with a single HPO term, we ranked the files by the result score; in combined cases by the provided statistical score as the program outputs a result score for each HPO term separately.

Below, we provide the raw result files for the different tools, showing the disease genes indicated by our users, and the associated rank and scores. *n/a* signifies that the disease gene was not found within the first 100 ranks. To protect our patient's privacy, we are not able to provide HPO terms or disease variants.

All tables are also available on our website as TSV files.

exomiser-cli-10.0.1 results

disease gene	algorithm	rank	score
NSD1	PhenIX	1	1
NSD1	HiPhive	1	0.763908
ARID1B	PhenIX	10	0.525093
ARID1B	HiPhive	5	0.728278
OPA1	PhenIX	1	1
OPA1	HiPhive	1	1
GRIN2B	PhenIX	108	0.168334
GRIN2B	HiPhive	209	0.507849
ARHGEF9	PhenIX	n/a	n/a
ARHGEF9	HiPhive	n/a	n/a
ZBTB18	PhenIX	n/a	n/a
ZBTB18	HiPhive	n/a	n/a
SLC6A8	PhenIX	6	0.651658
SLC6A8	HiPhive	3	0.715505
MYO5A	PhenIX	2	0.658431
MYO5A	HiPhive	3	0.70801
SETD5	PhenIX	n/a	n/a
SETD5	HiPhive	1	0.744129
SETBP1	PhenIX	5	0.493021
SETBP1	HiPhive	14	0.542526
ASXL3	PhenIX	n/a	n/a
ASXL3	HiPhive	n/a	n/a
CREBBP	PhenIX	1	1
CREBBP	HiPhive	1	0.705202
ARID1A	PhenIX	5	0.839204
ARID1A	HiPhive	16	0.618128
EPG5	PhenIX	n/a	n/a
EPG5	HiPhive	n/a	n/a
ARID1B	PhenIX	10	0.748092
ARID1B	HiPhive	2	0.661683
BIN1	PhenIX	113	0.188608
BIN1	HiPhive	342	0.504722
NEB	PhenIX	1	1
NEB	HiPhive	3	0.877591
ELAC2	PhenIX	15	0.554671
ELAC2	HiPhive	14	0.688782
NEB	PhenIX	4	0.756883
NEB	HiPhive	1	0.801883
ADAR	PhenIX	190	0.195306
ADAR	HiPhive	29	0.548876
ATP6	PhenIX	n/a	n/a
ATP6	HiPhive	n/a	n/a

TREX1	PhenIX	2	0.953872
TREX1	HiPhive	4	0.899042
SLC6A1	PhenIX	n/a	n/a
SLC6A1	HiPhive	51	0.705423
ACTA1	PhenIX	1	0.710311
ACTA1	HiPhive	3	0.635305
HGSNAT	PhenIX	2	0.64649
HGSNAT	HiPhive	8	0.713038
GMPPB	PhenIX	36	0.409751
GMPPB	HiPhive	30	0.547259
TWNK	PhenIX	22	0.588044
TWNK	HiPhive	89	0.578214
TNNI2	PhenIX	13	0.658202
TNNI2	HiPhive	16	0.736839
COQ8A	PhenIX	26	0.363836
COQ8A	HiPhive	10	0.592884
ITPR1	PhenIX	9	0.784141
ITPR1	HiPhive	5	0.853847
PDHX	PhenIX	1	0.887272
PDHX	HiPhive	1	0.925893
REEP1	PhenIX	85	0.412009
REEP1	HiPhive	156	0.522467
POMK	PhenIX	10	0.398121
POMK	HiPhive	n/a	n/a
SACS	PhenIX	2	0.965364
SACS	HiPhive	1	0.780765
ND6	PhenIX	n/a	n/a
ND6	HiPhive	n/a	n/a
HADHB	PhenIX	10	0.543718
HADHB	HiPhive	107	0.579905
ACAD9	PhenIX	6	0.526988
ACAD9	HiPhive	5	0.743163
SERAC1	PhenIX	1	0.768556
SERAC1	HiPhive	1	0.771277
HIBCH	PhenIX	27	0.327435
HIBCH	HiPhive	11	0.691884
EXOSC3	PhenIX	1	0.760529
EXOSC3	HiPhive	3	0.634591
RYR1	PhenIX	1	1
RYR1	HiPhive	5	0.942731
LMNA	PhenIX	1	1
LMNA	HiPhive	1	0.928971
PDHX	PhenIX	1	1
PDHX	HiPhive	1	0.965827
CC2D2A	PhenIX	1	0.713541
CC2D2A	HiPhive	7	0.762057

VIPAS39	PhenIX	1	0.940524
VIPAS39	HiPhive	2	0.749259
SETX	PhenIX	1	1
SETX	HiPhive	5	0.817083
PRPS1	PhenIX	n/a	n/a
PRPS1	HiPhive	n/a	n/a
RAB3GAP1	PhenIX	1	0.953336
RAB3GAP1	HiPhive	1	0.837482
SH3TC2	PhenIX	1	0.333732
SH3TC2	HiPhive	6	0.531488
DMD	PhenIX	1	1
DMD	HiPhive	1	0.994963
WASHC5	PhenIX	50	0.137456
WASHC5	HiPhive	3	0.907151
ATM	PhenIX	3	0.656113
ATM	HiPhive	6	0.770853
ETFDH	PhenIX	5	0.677087
ETFDH	HiPhive	8	0.708204
EIF2B3	PhenIX	1	0.886438
EIF2B3	HiPhive	2	0.78582
ALX1	PhenIX	21	0.480594
ALX1	HiPhive	45	0.609137
MPZ	PhenIX	1	0.449694
MPZ	HiPhive	3	0.645236
MFN2	PhenIX	1	1
MFN2	HiPhive	25	0.904534
AARS	PhenIX	2	0.613797
AARS	HiPhive	7	0.752707
IGHMBP2	PhenIX	4	0.405006
IGHMBP2	HiPhive	11	0.55081
PEX14	PhenIX	10	0.582961
PEX14	HiPhive	15	0.673583
BCS1L	PhenIX	1	0.988725
BCS1L	HiPhive	3	0.888703
ABCC6	PhenIX	1	0.873603
ABCC6	HiPhive	2	0.80331
C12orf65	PhenIX	1	0.718803
C12orf65	HiPhive	n/a	n/a
HDAC8	PhenIX	n/a	n/a
HDAC8	HiPhive	n/a	n/a
SMARCA4	PhenIX	n/a	n/a
SMARCA4	HiPhive	n/a	n/a
ACTB	PhenIX	7	0.63718
ACTB	HiPhive	16	0.538961
SMC1A	PhenIX	n/a	n/a
SMC1A	HiPhive	n/a	n/a

B3GLCT	PhenIX	1	1
B3GLCT	HiPhive	1	0.813255
TGFB2	PhenIX	1	1
TGFB2	HiPhive	3	0.931756
LTBP4	PhenIX	1	1
LTBP4	HiPhive	1	1
FBN1	PhenIX	1	1
FBN1	HiPhive	1	1
TGFB2	PhenIX	1	1
TGFB2	HiPhive	3	0.931756
SMAD3	PhenIX	1	1
SMAD3	HiPhive	3	0.858134
FBN1	PhenIX	1	1
FBN1	HiPhive	1	1
TGFBR2	PhenIX	1	1
TGFBR2	HiPhive	3	0.888418
TGFBR2	PhenIX	1	1
TGFBR2	HiPhive	3	0.888418
SMAD3	PhenIX	1	1
SMAD3	HiPhive	3	0.858134
ALDH18A1	PhenIX	1	1
ALDH18A1	HiPhive	1	1
EXOSC3	PhenIX	1	0.899412
EXOSC3	HiPhive	1	0.847262
RAB3GAP1	PhenIX	1	1
RAB3GAP1	HiPhive	1	0.848651
PRPS1	PhenIX	n/a	n/a
PRPS1	HiPhive	n/a	n/a
SETX	PhenIX	1	0.816198
SETX	HiPhive	6	0.806825
EIF2B3	PhenIX	6	0.471054
EIF2B3	HiPhive	32	0.578578
WASHC5	PhenIX	50	0.17337
WASHC5	HiPhive	1	0.870021
HSPG2	PhenIX	4	0.763745
HSPG2	HiPhive	17	0.641165
TNNI2	PhenIX	5	0.939636
TNNI2	HiPhive	3	0.92566
CHRNG	PhenIX	1	0.726079
CHRNG	HiPhive	2	0.812818
TTN	PhenIX	57	0.462641
TTN	HiPhive	73	0.565785
HADHA	PhenIX	1	0.809361
HADHA	HiPhive	3	0.625842
ECHS1	PhenIX	n/a	n/a
ECHS1	HiPhive	4	0.704007

SACS	PhenIX	2	0.710355
SACS	HiPhive	10	0.665523
HIBCH	PhenIX	21	0.408055
HIBCH	HiPhive	8	0.586799
HK1	PhenIX	1	0.39325
HK1	HiPhive	11	0.537688
EFEMP2	PhenIX	1	1
EFEMP2	HiPhive	1	1
PYCR1	PhenIX	1	1
PYCR1	HiPhive	1	0.957177
PYCR1	PhenIX	1	1
PYCR1	HiPhive	1	0.957177
HRAS	PhenIX	n/a	n/a
HRAS	HiPhive	n/a	n/a
NBAS	PhenIX	1	1
NBAS	HiPhive	1	1
GORAB	PhenIX	1	1
GORAB	HiPhive	1	1
NALCN	PhenIX	n/a	n/a
NALCN	HiPhive	n/a	n/a
SATB2	PhenIX	52	0.301952
SATB2	HiPhive	3	0.811406

eXtasy results

disease gene	rank	score
NSD1	22	3.04E-11
ARID1B	n/a	n/a
OPA1	20	596
GRIN2B	139	1.26E-08
ARHGEF9	219	3.47E-09
ZBTB18	1	
SLC6A8	44	2.81E-19
MYO5A	129	1.83E-09
SETD5	n/a	n/a
SETBP1	975	0.076636
ASXL3	n/a	n/a
CREBBP	9	1.44E-28
ARID1A	249	1.76E-12
EPG5	n/a	n/a
ARID1B	n/a	n/a
BIN1	2268	0.278484
NEB	n/a	n/a
ELAC2	n/a	n/a
NEB	n/a	n/a
ADAR	302	0.000294
ATP6	n/a	n/a
TREX1	n/a	n/a
SLC6A1	1214	0.027912
ACTA1	73	0.000608
HGSNAT	n/a	n/a
GMPPB	n/a	n/a
TWNK	1	
TNNI2	2	7.23E-09
COQ8A	n/a	n/a
ITPR1	n/a	n/a
PDHX	n/a	n/a
REEP1	n/a	n/a
POMK	n/a	n/a
SACS	958	0.012684
ND6	n/a	n/a
HADHB	26	564
ACAD9	52	0.000307
SERAC1	n/a	n/a
HIBCH	622	0.000639
EXOSC3	474	0.000603
RYR1	n/a	n/a
LMNA	11	2.67E-07
PDHX	11	2.98E-08

VIPAS39	1	
SETX	n/a	n/a
PRPS1	27	0.000207
RAB3GAP1	n/a	n/a
DMD	65	2.73E-05
WASHC5	n/a	n/a
ATM	n/a	n/a
ETFDH	97	6.52E-06
EIF2B3	1	1.80E-09
ALX1	387	0.0003
MPZ	98	306
MFN2	35	548
IGHMBP2	n/a	n/a
PEX14	1000	0.005375
BCS1L	80	408
ABCC6	77	4.33E-05
C12orf65	458	0.021386
HDAC8	770	0.000904
SMARCA4	n/a	n/a
ACTB	1	1.69E-19
SMC1A	2	1.88E-14
B3GLCT	n/a	n/a
EXOSC3	381	0.000404
RAB3GAP1	n/a	n/a
PRPS1	n/a	n/a
SETX	n/a	n/a
EIF2B3	56	1.16E-05
WASHC5	1	
HSPG2	39	2.81E-07
TNNI2	1	908
CHRNG	n/a	n/a
TTN	n/a	n/a
HADHA	7	1.97E-09
ECHS1	n/a	n/a
SACS	773	0.005968
HIBCH	678	0.000781
HK1	200	118
EFEMP2	106	0.4
PYCR1	n/a	n/a
PYCR1	160	356
HRAS	2	0.91
NBAS	323	0.25
GORAB	327	248
NALCN	n/a	n/a
SATB2	n/a	n/a

MutationDistiller results

disease gene	rank	score	topscore
NSD1	1	112.6319	112.6319
ARID1B	14	17.93709	54.14417
OPA1	2	7.563113	8.063113
GRIN2B	1	55.06609	55.06609
ARHGEF9	62	3.664438	77.4857
ZBTB18	58	3.664438	42.94717
SLC6A8	8	37.88197	66.52096
MYO5A	3	45.54908	48.63414
SETD5	31	23.07749	98.30192
SETBP1	2	12.18036	13.26585
ASXL3	49	15.38186	89.43976
CREBBP	3	80.97056	130.2281
ARID1A	1	103.7881	103.7881
EPG5	1	70.34067	70.34067
ARID1B	1	94.12933	94.12933
BIN1	2	28.67969	35.03408
NEB	1	56.41226	56.41226
ELAC2	7	13.19288	29.41884
NEB	5	23.62145	35.12638
ADAR	1	42.42425	42.42425
ATP6	3	29.65437	34.17371
TREX1	1	47.69625	47.69625
SLC6A1	18	9.764151	42.07963
ACTA1	1	36.22646	36.22646
HGSNAT	9	4.248784	19.23757
GMPPB	5	17.95807	50.65403
TWNK	14	10.88759	27.65856
TNNI2	5	16.72597	39.2495
COQ8A	7	14.96301	33.06244
ITPR1	8	25.95786	43.18598
PDHX	3	37.69727	42.21829
REEP1	24	6.003142	26.13097
POMK	14	2.322901	19.74893
SACS	1	48.11153	48.11153
ND6	2	35.83701	43.70047
HADHB	6	0.14963	18.51884
ACAD9	2	24.17498	46.21093
SERAC1	4	33.03232	50.18314
HIBCH	20	10.01568	39.82767
EXOSC3	7	17.28767	23.19486
RYR1	4	22.2207	25.67539
LMNA	3	31.07535	34.53004
PDHX	1	48.81932	48.81932

CC2D2A	1	12.78905	12.78905
VIPAS39	9	20.94097	56.16055
SETX	1	30.04922	30.04922
PRPS1	6	6.456883	21.72946
RAB3GAP1	5	24.01152	28.94409
SH3TC2	0	0	35.08977
DMD	1	41.94081	41.94081
WASHC5	5	17.45758	27.52063
ATM	13	0.71177	21.00033
ETFDH	17	9.786866	45.43582
EIF2B3	2	29.21946	29.30067
ALX1	6	16.434	26.07152
MPZ	3	0.14963	0.5
MFN2	3	13.14961	15.9222
AARS	5	0.185188	30.50832
IGHMBP2	1	14.53591	14.53591
PEX14	1	36.91173	36.91173
BCS1L	1	41.88399	41.88399
ABCC6	5	16.9613	27.5515
C12orf65	7	4.587224	17.23571
HDAC8	7	32.372	50.61073
SMARCA4	4	18.74492	24.46094
ACTB	2	43.22659	61.99797
SMC1A	11	15.87036	43.42287
B3GLCT	1	124.687	124.687
TGFB2	1	21.38557	21.38557
LTBP4	1	18.00114	18.00114
FBN1	1	21.38557	21.38557
TGFB2	1	21.38557	21.38557
SMAD3	1	21.38557	21.38557
FBN1	1	21.38557	21.38557
TGFBR2	1	21.88557	21.88557
TGFBR2	1	21.38557	21.38557
SMAD3	1	21.38557	21.38557
ALDH18A1	1	18.00114	18.00114
EXOSC3	3	29.83985	50.31187
RAB3GAP1	1	38.8249	38.8249
PRPS1	6	6.456883	21.72946
SETX	1	46.51053	46.51053
EIF2B3	20	0.0947	19.47399
WASHC5	3	20.12103	27.2652
HSPG2	2	23.53402	23.58726
TNNI2	1	16.72597	16.72597
CHRNG	1	16.81451	16.81451
TTN	7	12.54133	27.53579
HADHA	2	20.71916	23.19074

ECHS1	1	35.06387	35.06387
SACS	4	24.97822	28.43531
HIBCH	18	14.41142	44.22341
HK1	4	0.14963	15.9222
EFEMP2	1	18.00114	18.00114
PYCR1	1	18.00114	18.00114
PYCR1	1	18.50114	18.50114
HRAS	1	18.50114	18.50114
NBAS	1	18.50114	18.50114
GORAB	1	18.00114	18.00114
NALCN	17	14.58517	34.32006
SATB2	13	21.14687	60.20118
